# Large difference but high correlation between creatinine and cystatin C estimated glomerular filtration rate in Mesoamerican sugarcane cutters

**DOI:** 10.1136/oemed-2021-107990

**Published:** 2022-03-30

**Authors:** Axel Andersson, Erik Hansson, Ulf Ekström, Anders Grubb, Magnus Abrahamson, Kristina Jakobsson, Yiyi Xu

**Affiliations:** 1 School of Public Health and Community Medicine, University of Gothenburg Sahlgrenska Academy, Gothenburg, Sweden; 2 La Isla Network, Washington, District of Columbia, USA; 3 Department of Laboratory Medicine, Lund University, Lund, Sweden; 4 Occupational and Environmental Medicine, Sahlgrenska University Hospital, Gothenburg, Sweden

**Keywords:** kidney diseases, occupational stress, occupational health, climate, physical exertion

## Abstract

**Objectives:**

To explore the relationship between creatinine and cystatin C based estimated glomerular filtration rate (eGFR) in actively working sugarcane cutters.

**Methods:**

This cohort study included 458 sugarcane cutters from Nicaragua and El Salvador. Serum samples were taken before and at end of harvest seasons and analysed for creatinine and cystatin C. Chronic Kidney Disease Epidemiology Collaboration (CKD-EPI) formulas were used to calculate eGFRs based on creatinine (eGFR_cr_), cystatin C (eGFR_cys_) and both creatinine and cystatin C (eGFR_crcys_) at each time point. Bland-Altman plots and paired t-tests were used to compare the difference between eGFR_cr_ and eGFR_cys_, and the difference in eGFRs between before and at end of the harvest seasons.

**Results:**

The mean eGFR_cr_ was higher than eGFR_cys_ in both cohorts; absolute difference 22 mL/min/1.73 m^2^ (95% CI 21 to 23) in Nicaragua and 13 mL/min/1.73 m^2^ (95% CI 11 to 15) in El Salvador. Correlations between eGFR_cr_ and eGFR_cys_ were high, with r=0.69, 0.77 and 0.67 in Nicaragua at pre-harvest, end-harvest and cross-harvest, and r=0.89, 0.89 and 0.49 in El Salvador.

**Conclusions:**

Creatinine increases among heat-stressed workers reflect reduced glomerular filtration as estimated using eGFR_cys_, a marker independent of muscle mass and metabolism. The discrepancy between eGFR_cr_ and eGFR_cys_ may indicate reduced glomerular filtration of larger molecules and/or systemic bias in CKD-EPI performance in this population.

**Key messages**

**What is already known on this topic?**

- Heat-stressed sugar cane cutters in Mesoamerica suffer from kidney injury during harvest season and high rates of chronic kidney disease.
- The usefulness of serum creatinine for surveillance of kidney function in manual workers with high musculoskeletal loads may however be limited by its dependence of muscle mass increase and muscle injury.

**What this study adds?**

- Changes in creatinine correlated well with changes in cystatin C, supporting that glomerular filtration rate decreased substantially over a harvest season.
- Cane cutters had much lower estimated glomerular filtration rate (eGFR) based on cystatin C than eGFRs based on creatinine, raising not only the question about the validity of eGFR equations, but also whether the observed discrepancy may indicate pathophysiological changes affecting glomerular pore size.

**How this study might affect research, practice and/or policy?**

- There is a need to validate the available methods for eGFR calculation with measured GFR in Mesoamerican working populations.
- Other markers than creatinine for surveillance of kidney function and injury in heat-stressed workers at high risk of chronic kidney disease should be evaluated.

## Introduction

Chronic Kidney Disease of Non-Traditional Origin (CKDnT) has led to the premature death of tens of thousands in Mesoamerica, predominantly male agricultural workers.^1^ Hypotheses on CKDnT aetiology have focused on recurrent heat stress, but pesticide use, metal toxicity and infections have also been suspected.^2^ The heat stress hypothesis has been studied among harvest workers at several sugarcane mills, finding frequent and substantial serum creatinine increases both across the work shift and harvest season.^3–6^

Studies on CKDnT have hitherto, with very few exceptions, reported estimated glomerular filtration rate based on serum creatinine (eGFR_cr_). Serum creatinine is heavily dependent on muscle mass, which could be problematic when manual labourers are evaluated, with more muscle mass or muscle degradation leading to higher levels.^7^ Cystatin C, another biomarker for kidney function, is independent from muscle mass.^7 8^ However, analysis of cystatin C is more expensive and less accessible than creatinine in most countries, but has been recommended as a possible addition in prevalence studies.^9^

Comparisons between eGFR_cr_ and cystatin C based eGFR (eGFR_cys_) have been performed mainly in North American, European and Asian populations. Some studies show good agreement between measured GFR and Chronic Kidney Disease Epidemiology Collaboration (CKD-EPI) calculated eGFR_cr_ and eGFR_cys_,^10–12^ while other studies showed systematically higher eGFR_cr_ than eGFR_cys_.^13–15^ We are only aware of one study reporting lower eGFR_cr_ than eGFR_cys_, but the difference was small.^16^ This variation between studies may be a consequence of variations in the rate of production and excretion of creatinine and cystatin C,^17^ but also laboratory analytical variation.

There are very limited studies in Mesoamerica that have reported cystatin C^18^ or eGFR_cys_,^19^ which could be of interest to understand the mechanism of deteriorating kidney function and CKDnT in this population.

Recent studies suggested that a relatively lower eGFR_cys_ than eGFR_cr_ strongly predict mortality in several groups such as cardiovascular patients, healthy seniors and other adult patients.^20^ Owing to the difference in size between creatinine (molar mass 113 Da) and cystatin C (13.3 kDa), a lower eGFR_cys_ than eGFR_cr_ may reflect a selectively decreased elimination of relatively large molecules. This phenomenon has been described in the literature as ‘Shrunken Pore Syndrome’ (SPS).^21 22^ The mechanism for increased disease risk has been suggested to be due to reduced elimination of pro-atherogenic and inflammatory proteins.^23^

The aims of this study were first to compare the levels of eGFR_cr_ and eGFR_cys_, second to evaluate how cross-harvest changes in eGFR_cys_ correspond to the change in the more widely used eGFR_cr_, in Mesoamerican sugarcane cutters at risk of CKDnT.

## Methods and materials

### Study population

This study included two different cohorts from two sugarcane mills, Ingenio San Antonio in Chinandega, Nicaragua, situated on the lowland Pacific coast, and Ingenio El Ángel in Apopa, El Salvador, with operations both in the coastal lowlands and in the inland, 450 m above sea level. Both mills have participated in interventions aiming at reducing heat stress by improved access to water, rest and shade (WRS-intervention). Evaluations of the cohorts and the interventions have been reported in detail elsewhere.^6 24 25^ Although both cohorts include Mesoamerican sugarcane workers, there are differences in climate and working conditions, implementation of the WRS-intervention, general living conditions and home conditions between the two cohorts. We predetermine the larger Nicaraguan cohort as the main cohort and the smaller El Salvadoran cohort as the validation cohort. All analyses were performed separately.

The present study included 360 male burned cane cutters and seed cutters from Nicaragua, investigated 2018–2019,^25^ and 98 male burned cane cutters from El Salvador, investigated 2015–2016.^6^ The Nicaraguan workers had passed a pre-employment screening formally requiring them to have a serum creatinine value ≤1.3 mg/dL and absence of dysregulated diabetes, hypertension or hyperuricaemia. No pre-employment screening existed at the mill in El Salvador.

Fifty-nine workers dropped out during harvest in Nicaragua, and 31 dropped out in El Salvador, many of them due to decreasing kidney function. Drop out in El Salvador was also related to security problems during the study period.

### Data collection and biomarker analysis

In both cohorts, questionnaire data and morning blood samples were collected immediately before and at end of harvest (November and April, respectively). Blood samples were drawn by phlebotomists. Serum was separated, frozen at −77°C and shipped to Lund, Sweden, for analysis. Creatinine and cystatin C were analysed in serum at the Department of Clinical Chemistry of the Skåne University Hospital in Lund, Sweden, using the Cobas 701-instrument (Roche Diagnostics, Basel, Switzerland). Creatinine was measured using an IDMS-calibrated, enzymatic colorimetric method. Cystatin C was measured using a particle enhanced immunoturbidimetric assay, which was standardised against the international reference material ERM-DA471/IFCC. Samples from before and end of harvest from each cohort were analysed in the same session, in order to eliminate analysis batch effects introducing spurious cross-harvest eGFR changes. All analyses were performed in the same laboratory for both the Nicaraguan and Salvadoran samples but at different time points. The laboratory is accredited according to ISO 15189. The details of analysis were also reported previously.^6 24 25^

### Statistical methods

The CKD-EPI equations were used to calculate eGFR_cr_, eGFR_cys_ and eGFR based on both creatinine and cystatin C (eGFR_crcys_) from creatinine and cystatin C in serum.^26^ All subjects were classified as ‘non-blacks’. The absolute differences of eGFR_cr_ and eGFR_cys_ was calculated as eGFR_cys_−eGFR_cr_ and the ratio was calculated as eGFR_cys_/eGFR_cr_. The mean eGFR (eGFR_mean_) was calculated as the average of eGFR_cr_ and eGFR_cys_.

Paired t-test was used to analyse the difference between eGFR_cys_ and eGFR_cr_, and to make cross-harvest comparisons of eGFR_cys_ and eGFR_cr_. The correlation between eGFR_cr_ and eGFR_cys_ cross-harvest change was analysed using Pearson’s correlation test. Two outlier workers (one in each population) with physiologically implausible increases in eGFR_cys_ across harvest (>40 mL/min/1.73 m^2^) were excluded from cross-harvest correlation coefficient analyses. Bland-Altman plots were used to illustrate the difference between eGFR_cys_ and eGFR_cr_. All the statistical analyses were performed in each cohort separately, using IBM SPSS 26.0.

## Results

The basic characteristics of the study population are described in table 1. In Nicaragua, 200 of the 360 sugarcane cutters worked as burned cane cutters, and the rest as seed cutters. In El Salvador, all workers were burned cane cutters. The two populations had similar disadvantaged socioeconomic characteristics, but the Salvadoran cutters were slightly older (median 32 vs 28 years).

**Table 1:** Basic characteristics of study participants (male sugarcane cutters) from Nicaragua and El Salvador

		Nicaragua	El Salvador
Burned cane cutters, n (%)		200 (56)	98 (100)
Seed cutters, n (%)		160 (44)	0 (0)
Age, median (IQR)		28 (23–33)	32 (25–39)
Weight in kg, median (IQR)		64 (59–71)	65 (57–75)
Height in cm, median (IQR)		166 (161–171)	165 (162–170)
Serum creatinine mg/dL, median (IQR)	Before harvest	1.00 (0.88–1.12)	0.84 (0.78–0.96)
	End of harvest	1.00 (0.89–1.17)	0.88 (0.81–0.98)
Serum cystatin C mg/L, median (IQR)	Before harvest	1.10 (1.00–1.25)	0.92 (0.82–1.10)
	End of harvest	1.12 (1.00–1.25)	0.92 (0.82–1.21)
eGFR_cr_<60 mL/min/1.73 m^2^, n (%)	Before harvest	3 (1)	9 (9)
	End of harvest	10 (3)	9 (13)
eGFR_cys_<60 mL/min/1.73 m^2^, n (%)	Before harvest	55 (15)	14 (14)
	End of harvest	60 (20)	13 (19)
eGFR_crcys_<60 mL/min/1.73 m^2^, n (%)	Before harvest	15 (4)	10 (10)
	End of harvest	31 (10)	10 (15)

eGFR_cr_, estimated glomerular filtration rate based on creatinine; eGFR_crcys_, estimated glomerular filtration rate based on both creatinine and cystatin C; eGFR_cys_, estimated glomerular filtration rate based on cystatin C.

The different eGFR equations yielded different estimates of the proportion of workers with eGFR <60 mL/min/1.73 m^2^ in Nicaragua and El Salvador. 15% and 20% of Nicaraguans had eGFR_cys_<60 mL/min/1.73 m^2^ before and at end of harvest, respectively, while only 1% and 3% had eGFR_cr_ <60 mL/min/1.73 m^2^ at the same times. In the Salvadoran cutters, the proportion with eGFRs <60 mL/min/1.73 m^2^ were more similar, both before (14% for eGFR_cys_ vs 9% for eGFR_cr_) and at end of harvest (19% vs 13%).

**Figure 1:**
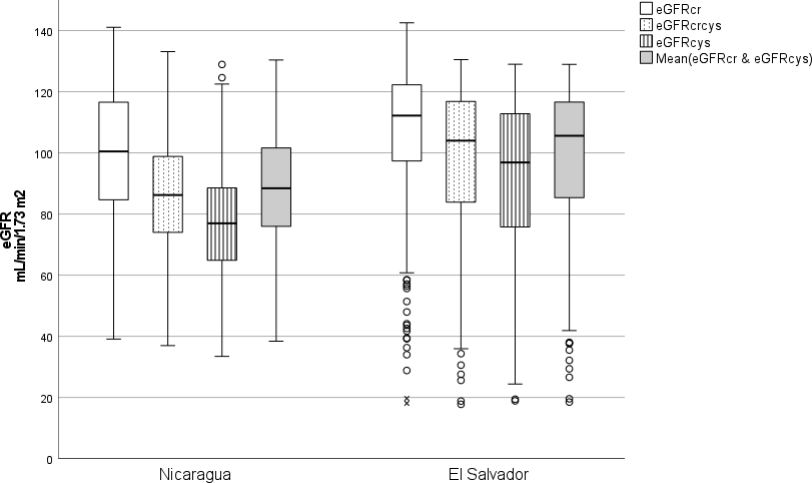
Boxplot showing estimated glomerular filtration rate based on creatinine (eGFR_cr_), cystatin C (eGFR_cys_), both creatinine and cystatin C (eGFR_crcys_), and the mean of eGFR_cr_ and eGFR_cys_. Measurements before and at end of harvest were combined in sugarcane workers from Nicaragua (n=360) and El Salvador (n=98), respectively. Circles depict outliers and crosses extreme outliers. The difference between all mean values were statistically significant (p<0.001; paired t-test).

**Figure 2:**
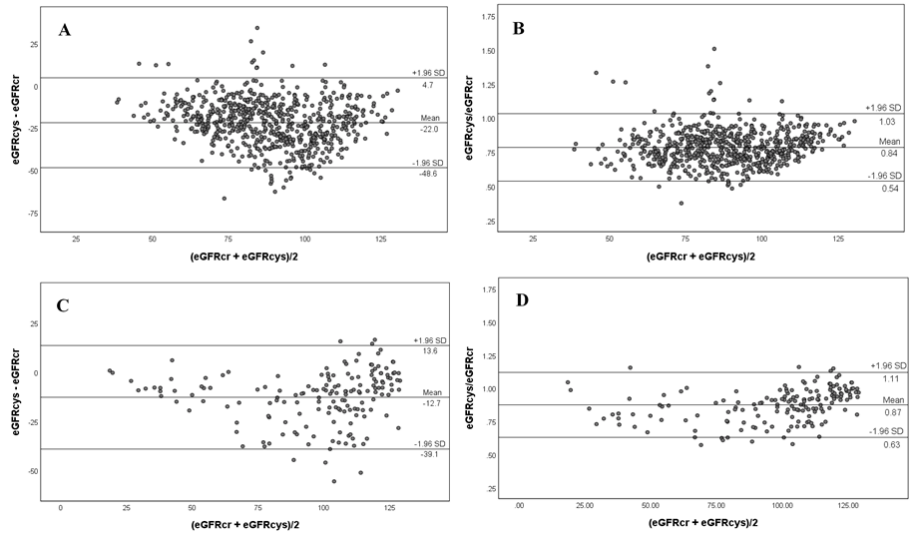
Bland-Altman plots showing estimated glomerular filtration rate based on creatinine (eGFR_cr_) and estimated glomerular filtration rate based on cystatin C (eGFR_cys_). The mean between eGFR_cr_ and eGFR_cys_ is on the x-axis, and either absolute difference or ratio between eGFR_cr_ and eGFR_cys_ on the y-axis. The eGFR values were calculated using the CKD-EPI formulas for creatinine and cystatin C. All values are expressed in mL/min/1.73 m^2^. (A) Absolute difference and (B) ratio for Nicaraguan sugarcane workers (n=360), sampled before and at the end of harvest. (C) Absolute difference and (D) ratio for Salvadoran sugarcane workers (n=98), sampled before and at the end of harvest. CKD-EPI, Chronic Kidney Disease Epidemiology Collaboration.

In the Nicaraguan cane cutters, eGFR_cys_ was much lower than eGFR_cr_, with a mean difference of −22 mL/min/1.73 m^2^ (95% CI −23 to −21), and an eGFR_cys_/eGFR_cr_-ratio of 0.79 (95% CI 0.78 to 0.80) (figure 1). In the Salvadoran cutters, the difference was −13 mL/min/1.73 m^2^ (95% CI −15 to −11) and the ratio 0.87 (95% CI 0.86 to 0.89). Bland-Altman plots showed triangular patterns with the largest differences in eGFR_cys_−eGFR_cr_ and eGFR_cys_/eGFR_cr_ at eGFR_cys_ and eGFR_cr_ mean values of approximately 90 mL/min/1.73 m^2^ in both study groups (figure 2).

Overall, all eGFR calculations (eGFR_cr_, eGFR_crcys_, eGFR_cys_ and eGFR_mean_) showed a similar decrease from before to end of harvest of about 3% (table 2). A similar pattern was seen in El Salvador, with a decrease of about 4%.

**Table 2:** Changes in estimated glomerular filtration rate based on creatinine, cystatin C and both creatinine and cystatin C over a harvest season in male sugarcane cutters from Nicaragua (n=360) and El Salvador (n=98)

	eGFR_cr_	eGFR_crcys_	eGFR_cys_	eGFR_mean_
**Nicaragua**				
eGFR before harvest (95% CI)	101 (99 to 103)	88 (85 to 89)	78 (76 to 80)	90 (88 to 92)
eGFR at end of harvest (95% CI)	98 (95 to 100)	85 (83 to 87)	76 (74 to 78)	87 (85 to 89)
Difference eGFR (95% CI)	−3 (−5 to −2)	−3 (−4 to −1)	−2 (−3 to −1)	−3 (−4 to −1)
% change (95% CI)	−3 (−5 to −2)	−3 (−5 to −2)	−3 (−4 to −1)	−3 (−4 to −2)
P value**	<0.001	<0.001	0.003	<0.001
**El Salvador**				
eGFR before harvest (95% CI)	105 (99 to 111)	98 (92 to 105)	93 (86 to 100)	99 (93 to 105)
eGFR at end of harvest (95% CI)	101 (94 to 108)	95 (88 to 101)	90 (82 to 97)	95 (88 to 102)
Difference eGFR (95% CI)	−4 (−6 to −2)	−4 (−6 to −2)	−3 (−6 to −1)	−4 (−6 to −2)
% change (95% CI)	−4 (−6 to −2)	−4 (−6 to −2)	−4 (−7 to −1)	−4 (−6 to −2)
P value**	<0.001	0.001	0.019	<0.001

*Significance evaluated with paired t-test.

eGFR, estimated glomerular filtration rate; eGFR_cr_, estimated glomerular filtration rate based on creatinine; eGFR_crcys_, estimated glomerular filtration rate based on both creatinine and cystatin C; eGFR_cys_, estimated glomerular filtration rate based on cystatin C; eGFR_mean_, mean between creatinine and cystatin C based estimated glomerular filtration rate.

**Figure 3:**
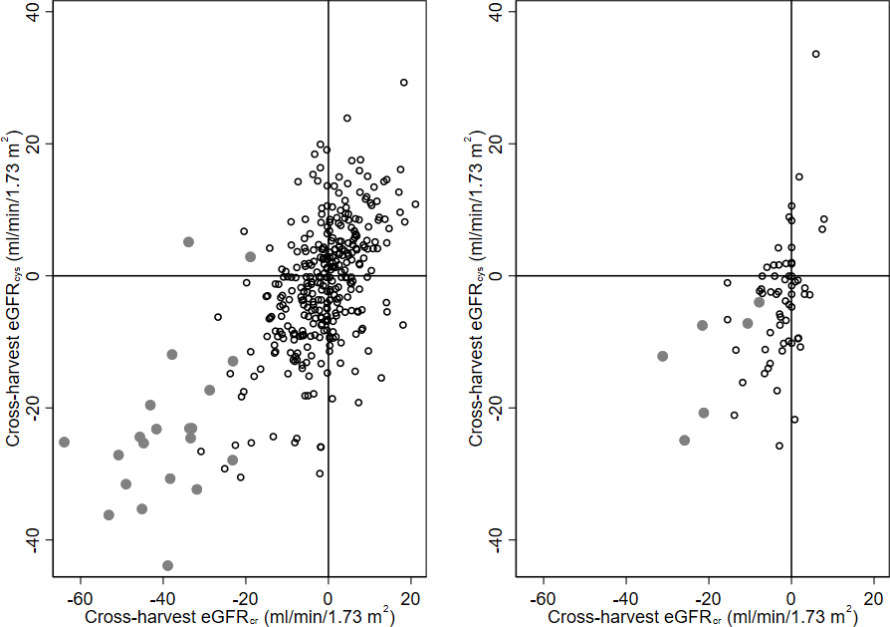
Correlation between cross-harvest change in eGFR_cr_ and eGFR_cys_ in male sugarcane workers from Nicaragua (left; n=360) and El Salvador (right; n=98). Grey dots indicate individuals with a creatinine increase by 0.3 mg/dL or 50% over harvest season. Two outliers with extreme increases in eGFR_cys_ (>40 mL/min/1.73 m^2^) are not shown. eGFR_cys_, estimated glomerular filtration rate based on cystatin C; eGFR_cr_, estimated glomerular filtration rate based on creatinine.

The correlation between the eGFR_cys_ and eGFR_cr_ among the Nicaraguan cane cutters before the harvest was 0.69 and at end of harvest 0.77. For the Salvadoran cutters, the correlation was 0.89 before and also at the end of harvest. The correlations between the cross-harvest differences for eGFR_cys_ and eGFR_cr_ were slightly lower, 0.67 for Nicaraguan cane cutters, and 0.49 for Salvadoran cutters (figure 3).

## Discussion

We found that Mesoamerican sugarcane cutters, a population at high risk of CKDnT, had generally lower eGFR_cys_ than eGFR_cr_, but the two GFR estimations were highly correlated.

Both eGFR_cys_ and eGFR_cr_ showed an average 3%–4% decrease over the harvest season. The correlations between cross-harvest eGFR_cr_ and eGFR_cys_ change were moderate to high, strongly indicating that creatinine increase among sugarcane cutters during harvest season does not merely reflect increased release of creatinine from muscles secondary to harvest work but reflects a true reduced glomerular filtration. Specifically, we found that workers with a creatinine increase of 0.3 mg/dL, which previously has been considered indicative of kidney injury,^3 25 27^ also had substantially reduced eGFR_cys_ and increased risk of markers of acute tubular injury in urine.^28^ This finding strengthens the use of serum creatinine increase as a potentially useful intermediary outcome in longitudinal studies aiming to understand CKDnT natural history and aetiology. However, further longitudinal studies are needed to establish the long-term consequences of such injury.

Our finding of lower eGFR_cys_ than eGFR_cr_ is similar to reports from a longitudinal study in a healthy general population from Nicaragua, where lower eGFR_cys_ than eGFR_cr_ was indicated but no actual difference was reported.^18^ In contrast, a biopsy study in 19 CKDnT patients with lower eGFR levels (mean 57 mL/min/1.73 m^2^) reported very similar levels of eGFR_cr_ and eGFR_cys_.^19^

The reasons behind the difference between eGFR_cys_ and eGFR_cr_ in our study are unclear. First, the validity of CKD-EPI equations varies both by ethnicity/race and region of residency.^29^ The CKD-EPI estimating equations were developed in North American and European populations and have, to our knowledge, not been validated against measured GFR in Mesoamerica. As the disparity between eGFR equations shown in this study may be due to poorly adapted eGFR equations for the Mesoamerican population, our observations call for future studies to validate or update the existing eGFR equations, through measured GFR, so that GFR can be estimated more accurately in this population. Second, CKD-EPI estimating equations have limited performance at early stages of reduced glomerular filtration,^7 30^ where we found a larger difference between eGFR_cys_ and eGFR_cr_.

Notably, a lower eGFR_cys_ than eGFR_cr_ may also reflect a selectively decreased elimination of relatively large molecules, denoted ‘shrunken pore syndrome’ (SPS),^21 22^ which might be an intermediary marker of kidney injury. Therefore, to protect the young, working Mesoamerican population who are most at risk of CKDnT, further understanding of the difference between eGFRs are needed. Consequently, identification and selection of the best biomarker (creatinine, cystatin C or both in combination) for eGFR estimation are highly relevant for disease screening. Again, studies investigating associations between measured GFR, eGFR_cr_ and eGFR_cys_ in Mesoamerican sugarcane cutters at GFR level intervals indicative of early disease stages (ie, CKD stage 2–3) are needed to inform patients, clinicians and researchers about the relevance of eGFR_cr_ and eGFR_cys_, and whether there is a benefit in measuring eGFR_cys_ instead of, or in addition to, eGFR_cr_. Given cystatin C is not regularly measured in some low-income countries due to the higher cost and lower availability in laboratories, the knowledge about the agreement between measured GFR and eGFRs, together with a cost–benefit analysis, are crucial for evaluating the importance and necessity of using cystatin C in Mesoamerican sugarcane workers and for guiding policy making and implementation.

As to limitations of the study, it is relevant to note that it was performed among male manual cane cutters, working full-time in a manually demanding job, and very few subjects had serum creatinine levels corresponding to eGFR_cr_ below 60 mL/min/1.73 m^2^ at the time of sampling. This is a limitation with respect to generalisation of the findings. Our results are not necessarily valid for women and for subjects with more markedly reduced glomerular function.

We believe several strengths in this study should be noted. First, sampling was systematically done before the workday,^6 25^ to avoid the daily cross-shift increase of creatinine found in sugarcane workers.^5^ Second, the potential risk of measurement bias was mediated by analysing the samples in the same laboratory, with baseline and end of harvest samples in the same batch. Third, similar findings in two independent cohorts, sampled and analysed separately, indicate a lesser risk of reporting chance findings.

At the Nicaraguan mill, cross-harvest eGFR change comparisons were performed in workers having passed a mid-harvest creatinine examination, which eliminated some workers from the cohort due to having a creatinine value above the pre-employment criteria of 1.3 mg/dL. Consequently, the eGFR change (especially eGFR_cr_ change) found in our Nicaraguan population was underestimated since workers with more severe increase in creatinine, thus possible larger reduction in eGFR_cr_ were excluded. Indeed, 11 out of 59 workers dropping out in the Nicaragua group said that they had left work due to kidney disease/creatinine elevation.^25^ Similar underestimated eGFR change may be the case in the Salvadoran workers too, since a number of cutters dropped out before end of harvest.^6^

In additional to a mid-harvest creatinine examination, there was a pre-employment screening at the Nicaraguan mill, but not at the mill in El Salvador. The screening procedure may have contributed to the large difference in proportion with eGFR <60 mL/min/1.73 m^2^ between eGFR_cr_ and eGFR_cys_ in workers from Nicaragua and El Salvador, as workers with eGFR_cr_ <60 mL/min/1.73 m^2^ are selectively removed from the Nicaraguan workforce. It should also be noted that the climate is hotter in the lowlands than in the inland, resulting in higher work-related heat stress in all the Nicaraguan cane cutters, and in the lowland Salvadorian cutters, while other Salvadorian cutters were from the inland.^6^ In summary, to find differences in eGFR between the cohorts is to be expected.

In conclusion, our repeated findings of decreased eGFR_cys_ over one harvest season in two cohorts of sugarcane cutters provides further evidence of a true reduction of glomerular filtration. However, the large difference between eGFR_cr_ and eGFR_cys_ in the Mesoamerican sugarcane cutters indicates currently insufficient knowledge on the true GFR and its estimations in this population. Our study highlights the need to validate the current estimation equations of eGFRs by using measured GFR in the target population, and the need for longitudinal studies to understand which GFR estimate provides most information on kidney disease prognosis in the population at high risk of CKDnT.

## Data Availability

The Nicaraguan data will be available upon reasonable request, while the Salvadorian data may be obtained from a third party. Contact the corresponding author for more information.
